# High-frequency stimulation–induced secondary hyperalgesia shifts temporal order judgment and increases precuneus nodal degree in healthy adults

**DOI:** 10.1371/journal.pone.0358552

**Published:** 2026-09-25

**Authors:** Yuki Morikawa, Michihiro Osumi

**Affiliations:** 1 Department of Rehabilitation, Heisei Memorial Hospital, Shijo-cho, Kashihara-city, Nara, Japan; 2 Graduate School of Health Sciences, Kio University, Umaminaka, Koryo-cho, Kitakatsuragi-gun, Nara, Japan; 3 Neurorehabilitation Research Center, Kio University, Umaminaka, Koryo-cho, Kitakatsuragi-gun, Nara, Japan; University of Pennsylvania Perelman School of Medicine, UNITED STATES OF AMERICA

## Abstract

Pain can influence attention and other higher-order cognitive processes. In experimental settings, acute pain has been linked to attentional prioritization of the painful body region. However, conventional experimental pain models often rely on brief, phasic nociceptive stimuli and are therefore less suited to examining spatial attention during a sustained, experimentally induced sensitized state. In this study, we examined whether high-frequency stimulation (HFS)-induced secondary hyperalgesia, as a model of acute experimental sensitization, was associated with changes in temporal order judgment (TOJ) and resting-state EEG-derived measures in healthy adults. Sixteen healthy adults (mean age, 28.5 years; seven women) underwent HFS of the forearm to induce secondary hyperalgesia. Spatial attention was assessed using a TOJ task, and resting-state EEG data were analyzed using source-level graph-theoretical measures. HFS increased pinprick pain sensitivity in the surrounding skin adjacent to the stimulation site. TOJ performance showed a significant shift in the point of subjective simultaneity (PSS) toward the HFS-treated side, indicating an attentional bias independent of subjective pain intensity. EEG analysis revealed a significant increase in nodal degree in the bilateral precuneus in the theta band, and this change was positively correlated with the PSS shift (ρ = 0.53, p = 0.036). These findings suggest that both behavioral attentional bias and short-term changes in resting-state EEG-derived measures can accompany acute experimentally induced sensitization. The present results should be interpreted within the scope of the HFS model as reflecting short-term modulation during an acute sensitized state rather than chronic pain-like reorganization.

## Introduction

Pain is crucial for survival as an alarm system against potential tissue damage [1,2]. At the same time, pain imposes a substantial cognitive load and influences attention and other higher-order functions [3–6]. Because attentional resources are limited, pain-related information is often prioritized over competing sensory inputs [1,2,7–10]. In experimental settings, acute pain in healthy individuals has been linked to an attentional bias toward the painful body region, consistent with the prior entry effect [10,11]. However, most conventional experimental pain models rely on brief, phasic nociceptive stimuli and are mainly suited to probing immediate responses under controlled laboratory conditions [12]. Such models are less suited to examining how spatial attention behaves during a more sustained, experimentally induced state of sensitization.

Central sensitization is characterized by enhanced responsiveness of nociceptive neurons and is implicated in many chronic pain conditions [13–17]. High-frequency electrical stimulation (HFS) is a well-established experimental method for inducing transient secondary hyperalgesia and short-lasting, LTP-like sensitization in healthy individuals [18–23]. In this sense, HFS provides a useful human experimental model for probing early sensitization-related processes under controlled laboratory conditions [24]. However, the effects of HFS are time-limited and do not reproduce the persistent supraspinal reorganization, long-term behavioral adaptation, or broader psychosocial changes that characterize chronic pain disorders [20,23–25]. Accordingly, HFS should be regarded as a model of acute experimentally induced sensitization rather than a model of chronic pain itself [24,25].

Even within such a transiently sensitized state, sensitization may influence both the behavioral allocation of spatial attention and the short-term intrinsic organization of brain networks [24]. At the behavioral level, lateralized attentional prioritization may be reflected in temporal order judgment (TOJ) performance through the prior entry effect [11]. At the neurophysiological level, experimentally induced sensitization may also be accompanied by short-term alterations in EEG-derived measures, including resting-state parameters [26] and evoked potentials [27].

On this basis, the present study examined whether HFS-induced secondary hyperalgesia was associated with changes in spatial attention and resting-state EEG-derived network measures in healthy adults. We hypothesized that HFS-induced secondary hyperalgesia would be associated with (1) a lateralized shift in temporal order judgment (TOJ) performance toward the sensitized side and (2) changes in EEG-derived nodal degree during the acute sensitized state.

## Methods

### Participants

Sixteen pain-free healthy adults (mean age, 28.5 years; standard deviation, 4.61 years; range, 22–38 years; seven women) participated in the study. Exclusion criteria were prior participation in experiments involving HFS; the presence of any psychiatric, neurological, cardiac, or chronic pain condition; regular use of psychotropic or analgesic medications; and any traumatic injury to the upper limbs within the past 6 months. All participants reported that they had not used any analgesic medication within the previous 12 h. All participants were right-handed, as assessed using the Flinders Handedness Survey [28].

The study was approved by the Ethics Committee of Kio University (No. R5-51) and registered with the University Hospital Medical Information Network Clinical Trials Registry (UMIN000053182). The study was conducted in accordance with the Declaration of Helsinki. Written informed consent was obtained from all participants prior to participation. Participants were recruited from June 1, 2024, to July 31, 2024. Given the exploratory nature of this study and the intensive experimental protocol, no formal a priori power analysis was performed, and the final sample size was determined by feasibility.

### High-frequency stimulation

**Fig 1:**
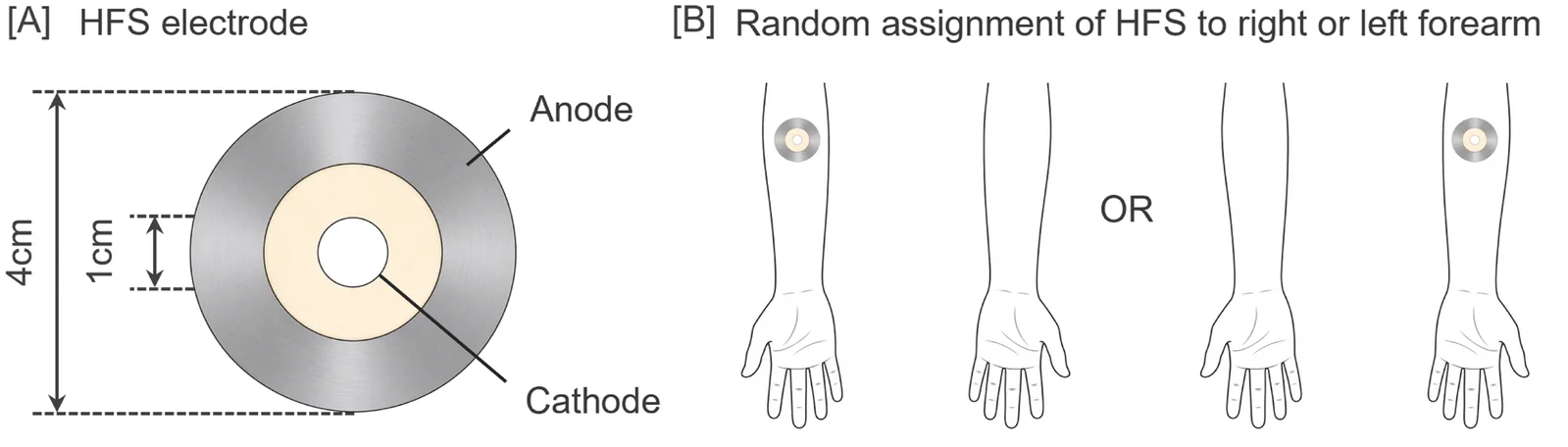
Schematic illustration of HFS. (A) Custom-built concentric electrode used for HFS. The electrode consisted of multiple blunt stainless-steel pins forming the cathode within a 1-cm-diameter circular area, surrounded concentrically by a ring-shaped anode with an outer diameter of 4 cm. (B) Placement of the HFS electrode on the volar forearm, 10 cm distal to the cubital fossa. Schematic adapted from previous reports [22,29].

HFS was delivered to the volar forearm using a constant-current stimulator (USE-200, UNIQUE MEDICAL Co., Ltd., Tokyo, Japan; Figs 1A and 1B). A custom-built concentric electrode based on a previous design [22] was used for stimulation. The electrode consisted of multiple blunt stainless-steel pins forming the cathode within a 1-cm-diameter circular area, surrounded by a ring-shaped stainless-steel anode.

The electrode was positioned 10 cm distal to the cubital fossa on the volar side of the forearm. HFS consisted of 10 trains of square-wave electrical pulses delivered at 100 Hz with a pulse width of 0.2 ms. Each train lasted 1 s and was followed by a 10-s intertrain interval. The stimulation intensity was individually set to 10 times the detection threshold and subsequently adjusted to ensure that pain intensity, as rated on a numerical rating scale (NRS; 0 = no pain, 10 = most intense pain imaginable), reached approximately 8. This stimulation protocol was adapted from a previously established human HFS protocol [22], with minor modifications to standardize perceived pain intensity at approximately 8 on the NRS. Participants were randomly assigned to receive HFS on the right or left forearm. To assess sensitization following the HFS procedure, we recorded pinprick pain intensity using an NRS (0 = no pain, 10 = most intense pain imaginable) before and after HFS. Pinprick stimuli were applied to the surrounding skin adjacent to the stimulation site using a blunt 0.7-mm-diameter needle (Chicago Medical Supply, LLC). Spatial mapping of the hyperalgesic area was not performed.

### Temporal order judgment task

**Fig 2:**
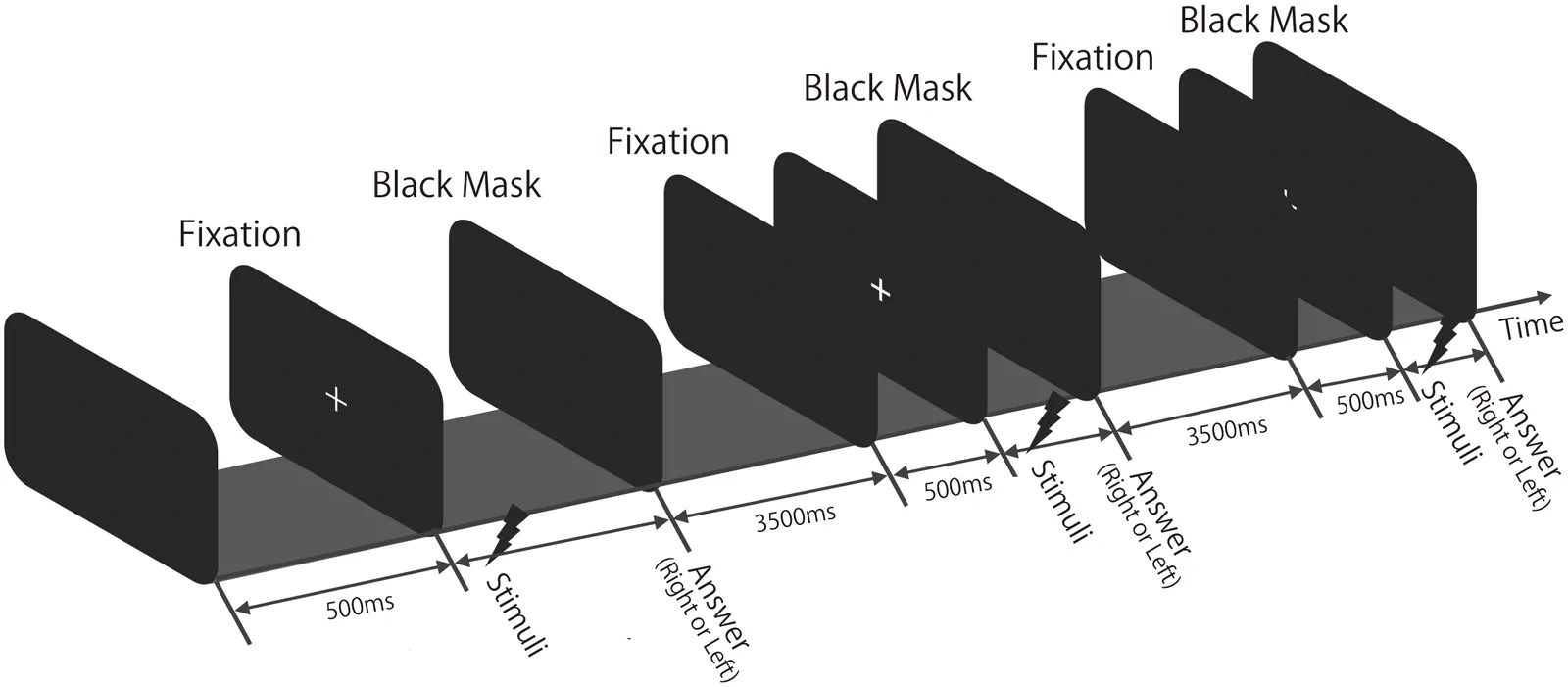
Trial structure of the TOJ task.

Electrical stimuli were applied to the volar side of both forearms using two constant-current stimulators (USE-200, UNIQUE MEDICAL Co., Ltd.; Fig 2). The electrodes were placed 10 cm distal to the cubital fossa of each forearm. Each stimulus consisted of a square-wave electrical pulse with a pulse width of 0.2 ms. The stimulation intensity was initially set at twice the sensory threshold for each forearm and subsequently adjusted so that participants perceived approximately equal intensity on both sides.

Each trial began with a black screen, and a fixation cross appeared after 0.5 s. Subsequently, a pair of electrical stimuli was delivered to the left and right forearms with a variable SOA defined relative to the HFS-treated side. Positive SOA values indicate that the HFS-treated side was stimulated first, whereas negative SOA values indicate that the non-HFS-treated side was stimulated first. Participants were asked to report which stimulus occurred first or second verbally. The order of trials was randomized, and a 3.5 s intertrial interval separated each trial. A total of 140 trials were administered.

During each trial, one stimulus was delivered to each forearm, and the order of stimulus presentation was determined by one of 14 stimulus onset asynchronies (SOAs): −480, −240, −120, −60, −30, −15, −5, 5, 15, 30, 60, 120, 240, and 480 ms. SOAs were recoded relative to the HFS-treated side; positive values indicated that the HFS-treated side was stimulated first, whereas negative values indicated that the non-HFS-treated side was stimulated first. The intertrial interval was set to 3.5 s.

Participants were asked to judge the temporal order of the two stimuli and verbally report which stimulus was presented first in a two-alternative forced-choice format. The question asked (i.e., “which stimulus came first” or “which stimulus came second”) was randomized across trials.

### Electroencephalography

EEG was recorded using a 64-channel EEG amplifier (ActiveTwo; BioSemi, Amsterdam, The Netherlands) at a sampling rate of 2048 Hz. Signal® Electrode Gel (Parker Laboratories, Fairfield, NJ, USA) was applied to each electrode to ensure adequate signal transmission. Electrode placement followed the standard 10–20 system, with reference electrodes configured as common mode sense and driven right leg electrodes, according to BioSemi’s EEG setup. The recorded EEG signals were amplified and digitized for each channel.

Resting-state EEG was recorded for 3 min both before and after HFS while participants were seated comfortably with their eyes closed. All recordings were conducted in an electrically shielded and sound-attenuated room with an ambient temperature maintained at a comfortable level. Participants were instructed to remain relaxed and minimize head and body movements during the recording session.

### Experimental procedure

**Fig 3:**
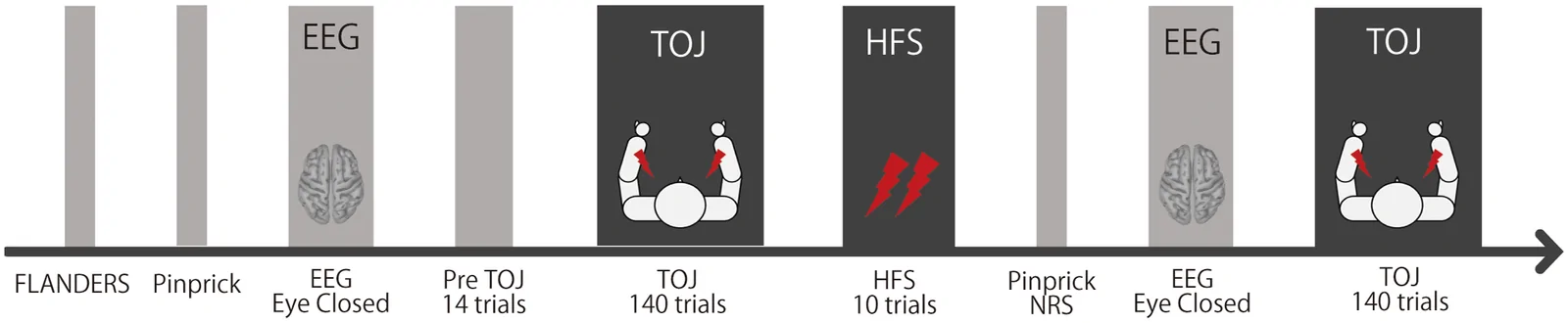
Experimental procedure.

During the experiment, participants were seated with both forearms resting on a table in a supinated position (i.e., palms facing upward), with the forearms positioned approximately 30 cm apart. A fixation point was placed approximately 50 cm in front of the participants along their midline (Fig 3).

At the beginning of the experiment, participants completed the FLANDERS handedness inventory, followed by an assessment of pinprick sensitivity using the NRS on the forearm scheduled to receive HFS. Resting-state EEG was then recorded for 3 minutes with eyes closed. Next, participants completed a practice session of the TOJ task consisting of 14 trials (one for each of the 14 SOA conditions). This was followed by 140 TOJ test trials (10 trials per SOA). Subsequently, HFS was delivered to one forearm in 10 trains, and pinprick sensitivity was reassessed in the surrounding skin adjacent to the stimulation site using the NRS. Another 3-minute eyes-closed resting EEG session was then conducted, followed by a second set of 140 TOJ trials (14 SOAs × 10 repetitions).

The Flinders Handedness Survey was administered to determine participants’ handedness. Pinprick stimuli were applied to the forearm designated for HFS, and pain intensity was assessed using an 11-point NRS, ranging from 0 (no pain) to 10 (most intense pain imaginable). Subsequently, resting-state EEG was recorded for 3 minutes while participants kept their eyes closed. To familiarize participants with the TOJ task, 14 practice trials were first presented (one for each SOA). This was followed by 140 formal trials (10 repetitions of each of the 14 SOAs). HFS was then delivered in 10 trains to the designated forearm, after which pinprick sensitivity was reassessed in the surrounding skin adjacent to the stimulation site using the NRS. The participants then underwent another 3-minute session of eyes-closed resting-state EEG. Finally, the TOJ task was repeated for 140 trials (10 repetitions for each SOA).

### Behavioral and EEG analyses point of subjective simultaneity

TOJ data were analyzed by plotting, for each participant, the proportion of trials in which the stimulus delivered to the HFS-treated forearm was perceived as occurring first, as a function of the stimulus onset asynchrony (SOA, in milliseconds). For participants who received left-sided HFS, SOA and response coding were recoded so that all psychometric functions were expressed relative to the HFS-treated side. Thus, positive SOA values indicated that the HFS-treated side was physically stimulated first, whereas negative SOA values indicated that the non-HFS-treated side was physically stimulated first. Response probability represented the probability of reporting the HFS-treated side as occurring first. A logistic (sigmoid) function was fitted to these data using MATLAB’s Curve Fitting Toolbox (R2024a, MathWorks Inc.):

y = 1 / [1 + exp(−a × (x − PSS))]

where x denotes SOA (ms), y denotes the response probability, and PSS represents the point of subjective simultaneity. The PSS was defined as the SOA corresponding to a 50% probability of reporting the HFS-treated side as occurring first on the fitted psychometric function.

### EEG preprocessing

The EEG data were first resampled at 256 Hz and subsequently subjected to a high-pass filter with a cutoff frequency of 1 Hz. To remove line noise at 60 Hz, we used a combination of the ZapLine algorithm (https://github.com/MariusKlug/zapline-plus) and the CleanLine plugin (https://github.com/sccn/cleanline/blob/master/eegplugin_cleanline.m), followed by a low-pass filter with a cutoff frequency of 100 Hz. Next, artifacts were automatically detected and corrected using the Adaptive Signal Reconstruction algorithm (https://github.com/t5i0m7/AASR). Channels identified as excessively noisy were temporarily removed and interpolated using a spherical spline method. The data were then re-referenced using the Reference Electrode Standardization Technique software (https://github.com/sccn/REST). Subsequently, the continuous EEG data were segmented into 2 s epochs with 50% overlap. Epochs were rejected if the standard deviation exceeded six times the average on any single channel or twice the average across all channels. Independent component analysis (https://github.com/sccn/eeglab/blob/develop/functions/popfunc/pop_runica.m) was then performed using the ICLabel algorithm (https://github.com/sccn/ICLabel) to classify components. Those identified as having less than 60% probability of being brain-related were automatically rejected.

### Source-level connectivity and graph analysis

EEG time series were reconstructed at the source level using standardized low-resolution brain electromagnetic tomography (sLORETA), which estimates a specific solution to the EEG inverse problem with zero localization error [30]. Localization in sLORETA is based on the standardization of current density estimates, providing a distributed representation of cortical sources. For each frequency band of interest (i.e., theta [4–7 Hz], alpha [8–13 Hz], and beta [14–30 Hz]), the preprocessed scalp EEG signals were band-pass filtered and projected into source space using the sLORETA algorithm. Source-level signals were extracted from 68 cortical regions of interest (ROIs) defined by the Desikan–Killiany atlas, which provides anatomically based parcellation of the cerebral cortex. No additional classification of ROIs into canonical functional networks was performed. Functional connectivity was assessed by computing amplitude envelope correlations (AECs) between each pair of ROI signals. Graph-theoretical measures were then derived from the resulting AEC-based connectivity matrices. In the present study, the primary graph-theoretical metric reported was nodal degree, defined as the number of connections a node has in the network [31,32]. Statistical analysis and interpretation in the present report were limited to nodal degree. These analyses were conducted using selected components of the DISCOVER-EEG pipeline (https://github.com/crisglav/discover-eeg), which provides modular tools for computing connectivity and network metrics from EEG data.

Participants were randomly assigned to receive HFS on either the right or left forearm (right: 7 participants; left: 9 participants). Source-level nodal degree was derived for each participant from the AEC-based connectivity matrices. Before statistical analysis, the nodal degree maps of the 9 participants who received left-sided HFS were flipped along the midline so that all cases were aligned relative to the stimulated side for group-level comparison. This side-normalization procedure was used because the primary aim of the present analysis was to examine effects relative to the stimulated side rather than anatomical left–right differences. Similar side-normalization procedures have been used in previous EEG studies of lateralized neurological conditions to align data according to the functionally relevant side for group-level analyses [33,34].

### Statistical analysis

To examine the relationship between the NRS ratings during HFS and pinprick-induced pain sensitivity, we performed a bivariate correlation analysis using Spearman’s rank correlation coefficient. Changes in the PSS before and after HFS were analyzed using paired t-tests. For each ROI, changes in AEC-based nodal degree before and after HFS were evaluated using a permutation test with 5000 randomizations. Multiple comparisons across ROIs were corrected using the false discovery rate procedure within each frequency band. A significance threshold of p < 0.05 was applied to all analyses.

In addition, Spearman’s rank correlation was performed to assess the relationship between the PSS shift index and the change in nodal degree of the bilateral precuneus. For the correlation analysis, we defined the PSS shift index as −(PSSpost − PSSpre), so that larger positive values indicated a greater shift toward relatively earlier perception of the HFS-treated side. The change in nodal degree was calculated as post-HFS minus pre-HFS and averaged across the bilateral precuneus. We also examined the correlation between the change in nodal degree and post-HFS pinprick pain ratings.

## Results

### HFS-induced sensitization and TOJ

**Fig 4:**
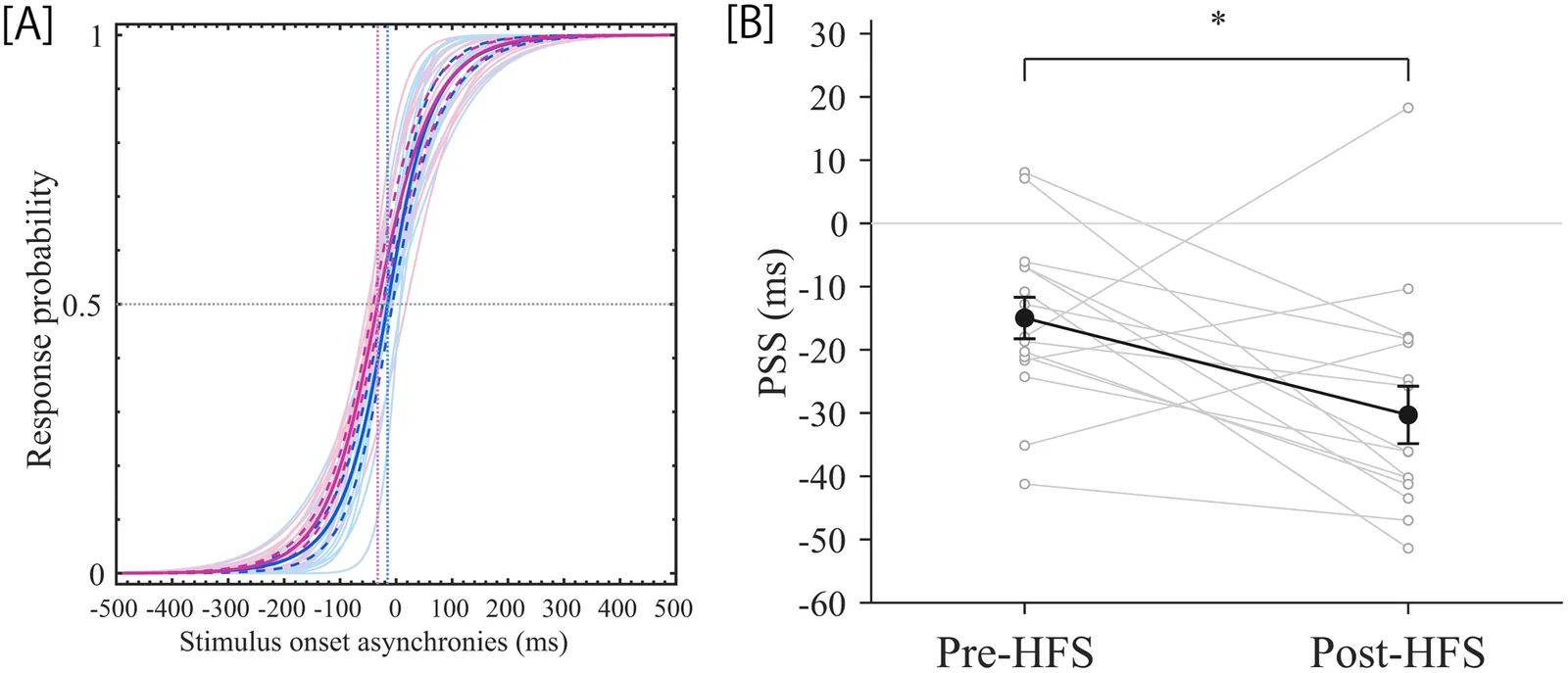
Changes in TOJ performance before and after HFS. (A) Psychometric functions fitted for each participant. The x-axis represents the stimulus onset asynchrony (SOA, ms), and the y-axis shows the probability of perceiving the HFS-treated side as occurring first. Positive SOA values indicate that the HFS-treated side was stimulated first, whereas negative SOA values indicate that the non-HFS-treated side was stimulated first. Curves in blue and pink correspond to pre-HFS and post-HFS conditions, respectively. The horizontal dotted line indicates a response probability of 0.5. Vertical dotted lines indicate the estimated PSS values for the pre-HFS and post-HFS conditions. (B) Individual and group-level PSS values before and after HFS. Gray lines indicate individual participants; black circles and error bars indicate the mean ± SEM. A significant negative shift in PSS was observed after HFS (paired t-test, p = 0.0161).

The electrical intensity during HFS was 13.63 ± 5.67 mA. HFS consisted of 10 trains, and the NRS rating at the 10th train was 7.75 ± 1.57. After HFS, pinprick pain ratings in the surrounding skin adjacent to the stimulation site were 3.25 ± 1.33 on the NRS, indicating increased pinprick sensitivity compared with baseline. No significant correlation was found between the NRS score during HFS and post-HFS pinprick pain (Spearman’s ρ = 0.25, *p* = 0.34). The mean PSS shifted significantly from −14.97 ± 13.12 ms before HFS to −30.30 ± 18.19 ms after HFS (mean difference [post–pre] = −15.33 ms, 95% CI −27.38 to −3.28 ms; t(15) = −2.712, p = 0.0161, Cohen’s dz = −0.678; Fig 4A and 4B), indicating a significant negative shift in PSS after HFS.

### Change in nodal degree after HFS

**Fig 5:**
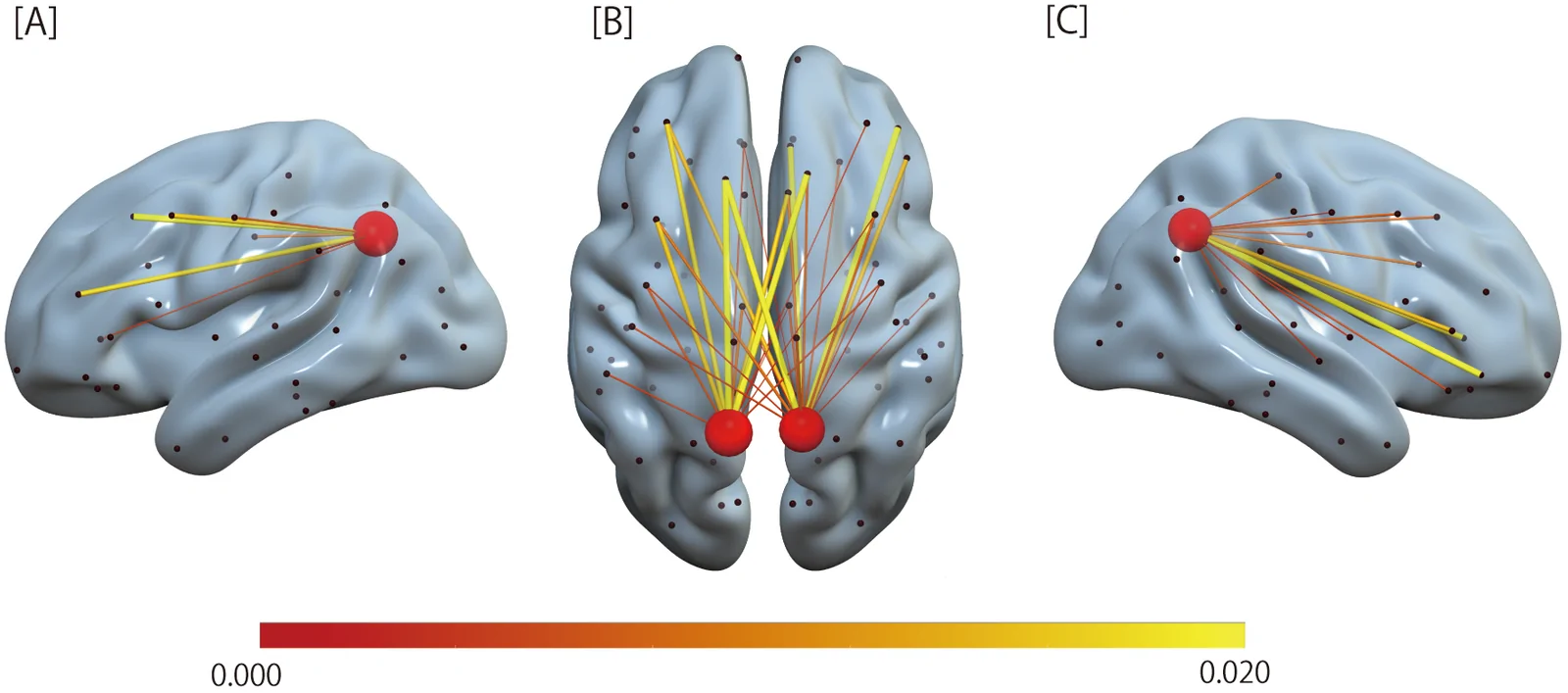
Nodal degree changes in the theta band following HFS. Compared with the pre-HFS condition, post-HFS data showed an increase in AEC-based nodal degree in the bilateral precuneus (shown in red nodes). (A) Left lateral view of the brain. (B) Superior view of the brain. (C) Right lateral view of the brain. The size and color of each node represent the absolute difference in nodal degree calculated as (post-pre) for each ROI. Statistical significance was determined using a permutation test with 5000 iterations and false discovery rate (FDR) correction for multiple comparisons (p < 0.05).

Source-level graph-theoretical analysis revealed significant changes in AEC-based nodal degree within the theta frequency band. A significant increase was observed in the bilateral precuneus after HFS (permutation test, 5000 iterations, false discovery rate-corrected p < 0.05; Fig 5). Furthermore, the PSS shift index was positively correlated with the mean AEC-based nodal degree change across the bilateral precuneus (post–pre; Spearman’s ρ = 0.53, p = 0.036; S1 Fig). However, no significant correlation was found between nodal degree change in the bilateral precuneus and post-HFS pinprick pain NRS score (Spearman’s ρ = 0.11, *p* = 0.68). Individual-level theta-band AEC-based nodal degree values and frequency-band-specific results for theta, alpha, and beta bands are shown in S2 Fig.

## Discussion

In the present study, HFS-induced secondary hyperalgesia was used as a model of acute experimentally induced sensitization in healthy adults. Within this transient sensitized state, we observed a significant negative shift in PSS and a significant increase in theta-band AEC-based nodal degree in the bilateral precuneus. Under the present SOA definition, the negative PSS shift is compatible with relatively earlier perception of the HFS-treated side. These findings should be interpreted cautiously within the physiological scope of the HFS model, which reflects a transient, experimentally induced, LTP-like sensitized state [24]. Taken together, the results suggest that short-lasting experimentally induced sensitization may be accompanied by both behavioral attentional bias and short-term neurophysiological modulation.

All participants showed increased pinprick sensitivity following HFS, consistent with the induction of an experimentally sensitized state. In the behavioral domain, the PSS shifted significantly in the negative direction after HFS. Under the present SOA definition, this shift is compatible with relatively earlier perception of the HFS-treated side. At the neurophysiological level, EEG analysis revealed a significant increase in AEC-based nodal degree in the bilateral precuneus. These findings suggest that acute experimentally induced sensitization can be accompanied by both behavioral and neurophysiological changes. Importantly, these findings should not be interpreted as evidence of persistent neural reorganization or chronic pain-like processes.

### Attentional bias during acute experimental sensitization

It should be noted that the pre-HFS PSS value was already negative, indicating a baseline temporal asymmetry in TOJ performance. Importantly, however, the main behavioral finding was the significant negative shift in PSS after HFS. Under the present SOA definition, in which positive values indicate that the HFS-treated side was physically stimulated first, this negative shift is compatible with relatively earlier perception of stimuli delivered to the HFS-treated side. Thus, even in the presence of baseline asymmetry, the direction of the pre-post change remains consistent with sensitization-related attentional prioritization.

This finding is consistent with previous research showing that acute noxious stimulation is associated with attentional bias toward the painful side, which can be explained by the prior entry effect, whereby stimuli delivered to attended locations are perceived earlier [11]. Our findings are also consistent with previous work suggesting that spatial attention can interact with experimentally induced sensitization [29]. Rather than indicating sustained neuroplastic change, this result likely reflects attentional prioritization within an acute sensitized state.

Despite the significant pre-post shift in PSS following HFS, there was no significant correlation between the PSS shift and subjective pain intensity as measured by the NRS. This dissociation suggests that the salience of the sensitized state, rather than pain intensity per se, may be sufficient to alter spatial attention. Pain captures attention because of its salience as a warning signal [1,7,35–37]. The attentional shift observed here may therefore reflect an implicit protective mechanism driven by nociceptive relevance rather than by explicit intensity ratings alone. Although the NRS captures conscious evaluation of pain intensity, attentional bias in the TOJ task may arise from lower-level processes involved in prioritizing behaviorally relevant somatosensory information.

This behavioral finding should nevertheless be interpreted with caution. Because the study did not include a sham or control condition, and all participants completed the same sequence of practice, baseline TOJ, HFS, and post-HFS TOJ, the observed pre-post shift in PSS cannot be attributed exclusively to HFS-induced sensitization. Practice effects, task-order effects, or regression to the mean may also have contributed to the observed change. Although the direction of the shift is compatible with sensitization-related attentional prioritization, causal attribution to HFS-induced sensitization remains limited by the absence of an appropriate control condition.

### EEG findings in the precuneus following HFS-induced secondary hyperalgesia

Our EEG analysis revealed a significant increase in AEC-based nodal degree in the bilateral precuneus following HFS-induced secondary hyperalgesia. The precuneus is a medial parietal region involved in internally oriented cognition, self-referential processing, and attentional integration [38–40]. In the present study, the increase in precuneus nodal degree may indicate short-term modulation of a region involved in internal information processing during the acute sensitized state. This finding should be interpreted as a region-specific change in AEC-based nodal degree, rather than as evidence of broader network-level reorganization.

Another notable finding was the significant positive correlation between the PSS shift index and the mean AEC-based nodal degree change across the bilateral precuneus. This association suggests that short-term neurophysiological modulation in the precuneus may be related to behavioral attentional bias during the acute sensitized state. Because resting-state EEG and TOJ performance were not recorded concurrently, this association should be interpreted as exploratory rather than as evidence of direct task-related neural–behavioral coupling. Importantly, this relationship was not explained by subjective pain intensity, suggesting that pain intensity per se may not be the primary driver of either the behavioral bias or the observed EEG change. Taken together, these findings suggest that acute experimental sensitization may be accompanied by short-term modulation of precuneus-related resting-state EEG-derived measures.

### Limitations

Several limitations should be considered when interpreting the present findings. First, although HFS-induced secondary hyperalgesia provides a useful model of acute experimentally induced sensitization, its transient nature does not capture the persistent neural, behavioral, and psychosocial complexity of chronic pain disorders. Accordingly, the present findings should not be interpreted as evidence that HFS reproduces the neural or behavioral features of chronic pain, but rather as evidence of short-term modulation during an acute sensitized state.

Second, secondary hyperalgesia was assessed indirectly using subjective pinprick pain ratings, and no spatial mapping of the hyperalgesic area was performed. Therefore, although increased pinprick sensitivity after HFS was observed, the induction of secondary hyperalgesia should be interpreted with appropriate caution.

Third, the study did not include a sham or control condition, and all participants underwent the same fixed sequence of practice, baseline TOJ, HFS, and post-HFS TOJ. Accordingly, the observed pre-post shift in PSS cannot be attributed exclusively to HFS-induced sensitization, and possible contributions of practice effects, task-order effects, or regression to the mean cannot be excluded. In addition, the negative pre-HFS PSS value indicates that baseline temporal asymmetry may have influenced the magnitude and interpretation of the observed pre-post shift.

Fourth, the sample size was modest, particularly for correlation analyses and graph-theoretical outcomes. The present study should therefore be regarded as exploratory, and the observed associations should be interpreted cautiously. With the current sample size, the study was primarily sensitive to relatively large effects; accordingly, the correlation between PSS shift and precuneus nodal degree, as well as the nodal degree findings, should be replicated in larger samples.

Fifth, the source-level nodal degree maps of the 9 participants who received left-sided HFS were flipped along the midline for side-normalized group-level analysis. Although this procedure allowed all cases to be aligned relative to the stimulated side, it does not preserve potential hemispheric asymmetries and may therefore obscure hemispherically specific effects. Accordingly, the spatially normalized EEG findings should not be interpreted as evidence of hemispherically specific cortical organization.

Finally, our EEG data were recorded at rest rather than concurrently with the post-HFS TOJ task. This temporal dissociation limits our ability to directly relate the observed EEG changes to task-specific attentional dynamics. Therefore, the observed correlation between precuneus nodal degree change and PSS shift should be interpreted as an exploratory association between measures obtained in different time windows, rather than as evidence of concurrent neural–behavioral coupling.

In addition, the present neurophysiological interpretation is based on AEC-based nodal degree alone. The analysis did not include global network metrics, density-controlled analyses, or classification of Desikan–Killiany ROIs into canonical functional networks. Therefore, the present findings do not establish global network reorganization and cannot determine whether the increased nodal degree in the precuneus reflected connections within a specific functional network or connections distributed across other cortical regions.

Consequently, although our findings suggest short-term modulation of precuneus-related resting-state EEG-derived measures associated with experimentally induced sensitization, a direct causal relationship between the EEG findings and behavioral attentional bias remains to be determined. Future studies using task-related electrophysiological measures during TOJ performance, together with broader network-level analyses and explicit functional network classification, will be important for clarifying the temporal and functional relationship between brain activity and attentional modulation in acute sensitized states.

## Conclusion

In healthy adults, HFS-induced secondary hyperalgesia was associated with a shift in PSS toward the sensitized side and an increase in theta-band nodal degree in the bilateral precuneus. The association between PSS shift and precuneus nodal degree change suggests that acute experimentally induced sensitization may be associated with both spatial attention bias and resting-state EEG-derived measures. These findings should be interpreted within the physiological scope of the HFS model, which reflects a transient, experimentally induced, LTP-like sensitized state rather than chronic pain itself [24]. Accordingly, the present results provide evidence of short-term behavioral and neurophysiological modulation during acute experimental sensitization.

## Supporting information

S1 Fig Association between PSS shift and precuneus nodal degree change. Scatter plot showing the relationship between the PSS shift index and the mean AEC-based nodal degree change across the bilateral precuneus. The PSS shift index was defined so that larger positive values indicated a greater shift toward relatively earlier perception of the HFS-treated side. Each point represents one participant. Spearman’s correlation coefficient and p-value are reported in the Results. The fitted line and shaded confidence interval are shown for visualization only.
(TIF)

S2 Fig Individual-level and frequency-band EEG results. (A) Individual-level theta-band AEC-based nodal degree values in the bilateral precuneus before and after HFS. Gray lines indicate individual participants; black circles and error bars indicate the mean ± SEM. (B) AEC-based nodal degree changes (post–pre) in the bilateral precuneus across theta, alpha, and beta frequency bands. Each gray circle represents an individual participant. Black circles and error bars indicate the mean ± SEM. No significant FDR-corrected nodal degree changes were observed in the alpha or beta bands.
(TIF)
